# CFD study of the water production in mature heavy oil fields with horizontal wells

**DOI:** 10.1371/journal.pone.0258870

**Published:** 2021-10-25

**Authors:** Andrés Pinilla, Miguel Asuaje, Camila Pantoja, Luis Ramirez, Jessica Gomez, Nicolás Ratkovich

**Affiliations:** 1 Department of Chemical Engineering, University of Los Andes, Bogotá, Colombia; 2 Frontera Energy, Bogotá, Colombia; China University of Mining and Technology, CHINA

## Abstract

Excessive water production in mature heavy oil fields causes incremental costs, energy consumption, and inefficiency. Understanding multiphase flows near the wellbore is an alternative to improve production efficiency. Therefore, this study conducts a series of numerical experiments based on the full set of the Navier-Stokes equations in 3D to simulate multiphase flows in porous media for heavy oil production horizontal wells. The solution given by this advanced mathematical formulation led to the description of the movement of the fluids near the wellbore with unprecedented detail. A sensitivity analysis was conducted on different rock and fluid properties such as permeability and oil viscosity, assuming homogeneous porous media. The influence of these parameters on the prediction of the breakthrough time, aquifer movement, and the severity of water production was noticed. Finally, the numerical model was verified against field data using two approaches. The first one was conducting a history match assuming homogeneous rock properties. In contrast, the second one used heterogeneous rock properties measured from well logging, achieving a lower deviation than field data, about 20%. The homogeneous numerical experiments showed that the breakthrough occurs at the heel with a subsequent crestation along the horizontal well. Moreover, at adverse mobility ratios, excessive water production tends to happen in water connings at the heel with an inflow area less than 1% of the total inflow area of the completion liner. Different aquifer movement dynamics were found for the heterogeneous case, like the breakthrough through multiple locations along the horizontal well. Finally, critical hydraulic data in the well, such as the pressure and velocity profiles, were obtained, which could be used to improve production efficiency. The numerical model presented in this study is proposed as an alternative to conducting subsurface modeling and well designs.

## Introduction

The production of heavy oil is challenging, and current research aims to improve its efficiency. Hydrocarbons are stored in porous rocks as oil and natural gas, and many times they interact with water in the form of aquifers. Multiphase flow in porous media derives from this interaction. Lenormand et al. (1988) proposed three multiphase flow patterns in porous media: stable displacement, capillary fingering, and viscous fingering (VF) [1]. These patterns are defined by the capillary number and the mobility ratio and tell if capillary or viscous forces dominate the displacement. The stable displacement is the most efficient, characterized by being the piston-like flow pattern. The capillary fingering regime occurs when the capillary forces are more significant than the viscous ones. It is characterized by having a relatively unstable interface between the displacing and displaced fluids. Small finger-like patterns can be distinguished at the interface, and oil can be bypassed because of this phenomenon. Finally, VF occurs when the viscous forces overcome the capillary ones, and it is the most inefficient displacement mechanism of the three. At the macro or reservoir scale, this phenomenon is the water channeling, cresting, and coning. VF is typical in heavy oil reservoirs due to the adverse mobility ratio and unfavorable capillary number. Theoretically avoidable by reducing the production rate to a minimum, but impossible for supplying the increasing energy demand of the modern world, which still relies on oil as the primary source of energy [2].

During VF, the interface is unstable, characterized by having finger-like patterns with well-established displacement mechanisms such as finger-birth, tip splitting, side branching, and shielding [3–5], leading to the dominance of several main fingers capable of channeling all the displacing water. This channeling has severe economic and environmental implications. On the one hand, water production can increase so dramatically in heavy oil reservoirs that the water-cut can easily reach values above 90% [6–9]. This implies an increasing operational cost, especially in lifting, which can grow exponentially from water-cuts above 80% [10], along with the extra costs for water management, treatment, reinjecting, and disposal. On the other hand, after the breakthrough, water production increases dramatically, displacing oil production. Consequently, energy consumption increases with adverse economic effects, such as reducing the energy return over investment, which measures the energetic efficiency of oil production [11]. Ultimately, the consequent increase in emissions and the worsening carbon footprint reveals the inefficiency of producing oil at high water-cuts.

With this unfortunate context, there is an increasing interest in understanding, comprehending, and predicting the multiphase flow in porous media to develop new and more efficient technologies and operations to mitigate the consequences previously exposed. The prediction of unstable displacements has been addressed using several approaches, like numerical simulations. One of them uses models based on the Diffusion-Limited Aggregation (DLA) method, which takes advantage of VF’s fractal growth [12–14]. Some of the earliest studies using this approach were conducted by Paterson [12] and Nittmann et al.[13], who demonstrated the possibility of simulating VF and its fractal growth. Other researchers like Tang & Wei [15] or Zhang et al.[16] have proposed modifications to the DLA method to consider the interface’s influence over the displacement, something that the original DLA formulation does not.

On the other hand, Tian & Lu [17] showed the possibility of using this method in 3D simulations, even considering the porous domain based on a pressure-oriented rule methodology, which relies on a previous numerical solution of the pressure field by a Lattice-Boltzmann simulation in the porous media. Unfortunately, the DLA method has been questioned for the modeling of multiphase flow in porous media. It is argued that the movement of the fluids is given by a stochastic random walk rather than the physical flow in porous media [18]. Also, because the measured fractal dimension in Hele-Shaw cells and porous media does not correspond to the fractal dimension of DLA [19] (1.7 *in* 2*D*, 2.45 *in* 3*D*).

Other modeling tools used to predict the multiphase flow in porous media are based on the Representative Elementary Volume, like the reservoir simulators. These codes are widely used and have been the most popular option to conduct numerical reservoir studies due to their capabilities of predicting up to compositional displacements in a relatively short time for the characteristic large length and time scales of oil reservoirs. Some studies found in the literature on reservoir simulators have addressed the channeling and cresting of water in horizontal wells, a macroscale consequence of VF [20–24]. However, they have not been shown to simulate the unstable displacement front as the physical models they are based on do not attempt. On the contrary, several studies have overcome this issue by implementing new mathematical models to scale up and consider the influence of VF. For example, Luo et al. [25–27] implemented the Effective Fingering Model, capable of considering VF effects in heavy oil displacements driven by water flooding and polymer flooding. The model developed by Luo et al. showed that these simulators could consider the effects of VF, having quantitative fair agreement compared to experimental and field data. Even with the qualitative description of the unstable water displacement front at the reservoir scale on large grids.

The last approach used to predict the unstable displacement dynamics is based on the conservation equations of fluid flow, like Computational Fluid Dynamics (CFD) codes. With this approach, several researchers have conducted numerical studies either at the pore-scale [28–38] or the continuum scale [39–52]. The difference between them is that the simulation at the pore-scale considers the porous geometry of the system. In contrast, in the continuum scale, the porous geometry is not considered explicitly. Instead, a value of porosity and permeability is used at each grid cell, similarly to the reservoir codes. Nevertheless, most of the studies using these approaches have been restricted to 2D systems, with few exceptions addressing the 3D dynamics of unstable displacements [31,38,39,45,51,52]. Although these models’ advantages are the possibility of explicitly simulating VF, either at the pore and continuum scale for miscible and immiscible displacements, the major drawback is that they have been limited to small length scales and 2D systems.

To fill the gap previously exposed, where the numerical models based on the conservation equations of fluid flow are now feasible with modern computational capabilities, this study, for the first time, proposes a CFD model for simulating unstable displacements for heavy oil horizontal wells with bottom aquifers, at the continuum scale in 3D. The numerical model simulates heavy oil-water flow in homogeneous and heterogeneous media, and it is verified against field data from a heavy oil well in Colombia. This study demonstrates the possibility of conducting near-wellbore simulations based on CFD, which can offer a high level of detail on modeling the aquifer’s unstable movement and calculating the breakthrough event, production rates, velocity, and pressure profiles inside the well, among others. The methodology and results presented in this study could help future researchers who want to exploit CFD models’ capabilities to improve and develop new technology for optimizing heavy oil production. For example, testing new production schemes or implementing completion technologies, like Inflow Control Devices.

## Methodology

A previous study demonstrated that CFD codes could explicitly simulate heavy oil-water flow, even emulating VF and its characteristic dynamics in coreflood experiments [53]. For the current study, the numerical model was extrapolated to a heavy-oil production well to study the bottom aquifer’s unstable displacement and breakthrough event near the wellbore. This study was divided into two parts. First, a parametric analysis was conducted to determine if the CFD model could simulate aquifer movement near the wellbore for different fluid and rock properties. In contrast, the second part was the verification of the numerical model conducting a history match. In this case, a heavy oil well from Colombia was simulated, considering homogeneous and heterogeneous rock properties, comparing the displacement differences near the wellbore. The homogeneous rock properties were measured from core sampling, while the heterogeneous properties were measured from the well logging acquisition. The CFD code used was the commercial software STAR-CCM+ v15.04. The simulations were run on a computer with an 8 core Intel Xeon E5-2690 processor and 64 GB of RAM. For the computational costs, each simulation took approximately 12 days to complete.

### Heavy oil well data

#### Sensitivity analysis on the influence of different fluid and homogeneous rock properties

This subsection describes the heavy oil production horizontal well used to conduct this research. As mentioned before, the first part of the study was to determine if the multiphase flow in porous media near the wellbore could be modeled in 3D based on the conservation equations used in CFD codes. Therefore, as the first approach, homogeneous porous media was considered. A sensitivity analysis on the rock and fluid properties was conducted to determine the feasibility of using this approach to model heavy oil horizontal wells.

**Fig 1:**
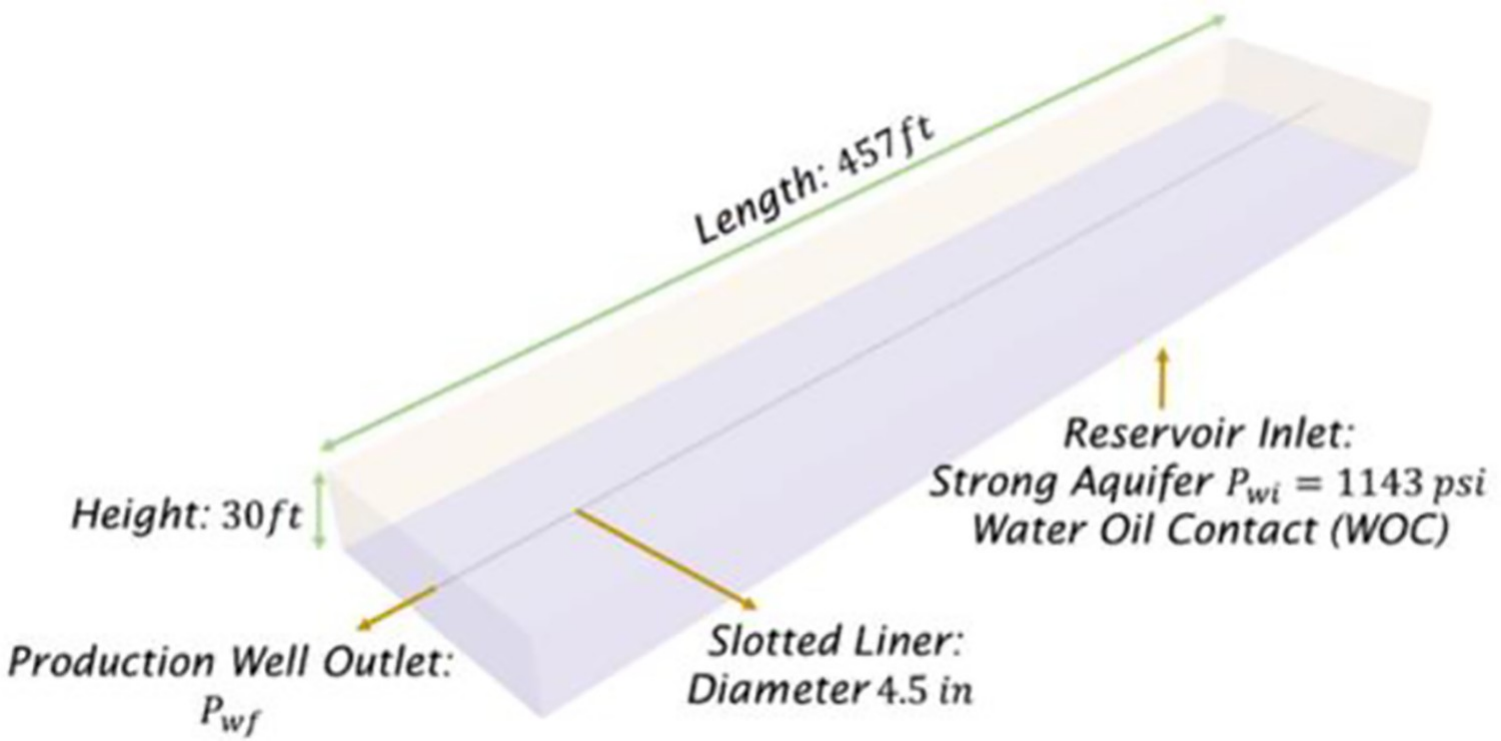
Domain geometry, initial, and boundary conditions used to conduct the numerical study.

A mature heavy oil well located in the Llano Basin, Colombia, was considered for this study. This well is presented in Fig 1, which summarizes the main dimensions, boundary conditions, and completion used. The horizontal well has a bottom aquifer with a reservoir pressure of *P*_*wi*_ = 1143*psi* and a horizontal liner of 4.5*in* in diameter. Additionally, Table 1 summarizes the primary petrophysical data from the reservoir in terms of the fluid physical properties, well dimensions, and rock properties. It must be pointed out that the rock properties were measured from core sampling and were initially assumed as homogeneous in the numerical model.

**Table 1:** Physical properties obtained from field measurements and used in the CFD reservoir-scale model.

Reservoir, well dimensions, and aquifer pressure	
Well length(*ft*)	457
Completion diameter (*in*)	4.5
Completion type	Slotted Liner
Slots per foot	224
Aquifer pressure *P*_*wi*_ (*psi*)	1143

Finally, a sensitivity analysis was conducted varying the oil and rock physical properties to determine if the numerical model could predict the fluid dynamics’ differences near the wellbore due to these variations. For example, the displacement stability is strongly influenced by variations in the permeability and the phases’ viscosity contrast. Both variables ultimately affect the mobility ratio and, consequently, the strength of the viscous forces. Therefore, Table 2 presents the matrix of more than 25 numerical experiments for this sensitivity analysis. It must be pointed out that this sensitivity analysis was conducted considering as base parameters the same ones presented in Table 3. Therefore, the rock and fluid physical properties and the initial conditions presented in Table 3 were kept constant during the numerical experiments of Table 2. It must be pointed out that the sensitivity analysis considered homogeneous, or uniform, properties in the whole domain.

**Table 2:** Summary of numerical experiments considered for the sensitivity analysis. The base parameters for each numerical experiment were kept constant to those presented later in Table 3.

Parameter	Base Data	Values					
**Porosity (%)**	32	10	20	30	40	50	-
**Permeability (*d*)**	2	0.01	0.1	1	10	100	
**Oil Viscosity (*Pa*∙*s*)**	0.109	0.001	0.01	0.1	1	10	100
**Density (*kg*/*m*** ^**3**^ **)**	972.50	992.02	978.04	965.05	952.06	939.08	927.09
***S***_***wi***_ **(%)**	52	0	10	20	30	40	-

**Table 3:** Physical properties obtained from field measurements and used in the CFD reservoir-scale model.

Fluid Properties	
Parameter	Value
Oil Viscosity (*Pa*∙*s*)	0.109
Oil Density (*kg*/*m*^3^)	972.5
Water Viscosity (*Pa*∙*s*)	1*x*10^−3^
Water Density (*kg*/*m*^3^)	1000
Surface Tension Oil-Water (*N*/*m*)	0.034
Contact angle (°)	30
**Reservoir, well dimensions, and aquifer pressure**	
Oil Water Contact (*TVD*−*ft*)	2999
Top basal sands (*TVD*−*ft*)	2969
Well length(*ft*)	457
Completion diameter (*in*)	4.5
Completion type	Slotted Liner
Slots per foot	224
*P*_*wf*_ (*psi*)	1143
**Rock properties and saturation conditions for the homogeneous case**	
*S*_*wi*_	0.520
*S*_*wir*_	0.117
*S*_*or*_	0.423
Porosity (−)	0.321
Permeability (*d*)	2
**Rock properties and saturation conditions for the heterogeneous case**	
*S*_*wi*_ (*min*−*max*)	0.17−0.66
Porosity (−)(*min*−*max*)	0.105−0.316
Permeability (*d*)(*min*−*max*)	0.006−14.400

#### Analysis on the influence of homogeneous and heterogeneous rock properties

After proving that CFD models can describe the aquifer movement in porous media and predict the changes in the displacement stability at different oil and rock physical properties, it is verified with a history match using field production data. For this purpose, two study cases are considered and compared: a homogeneous and heterogeneous case.

**Fig 2:**
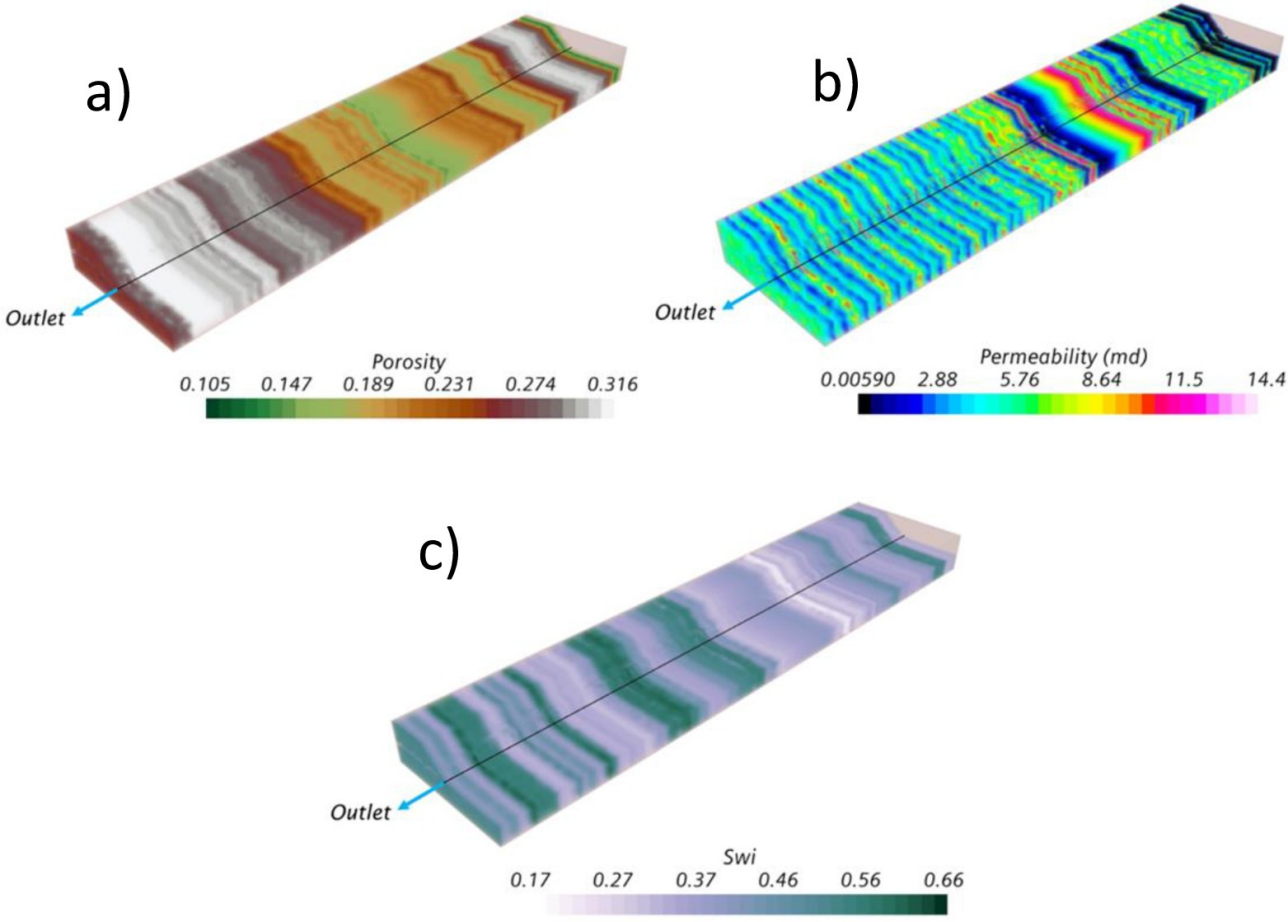
Heterogeneity profiles of a) porosity, b) permeability, and c) initial water saturation in the studied heavy oil reservoir.

The homogeneous case considered homogeneous rock properties acquired from core sampling. This assumption means that uniform values were used in all domain locations, not only rock properties but also the initial water saturation. In contrast, the heterogeneous case considered a vertical heterogeneous rock profile acquired from well logging measurements every 0.5*ft*. For both cases, the well dimensions, completion, and boundary conditions are the same ones previously presented in Table 1 and Fig 1. Table 3 presents the additional information related to the rock and fluid physical properties. Finally, Fig 2 presents the heterogeneous rock profile in terms of permeability, porosity, and initial water saturation. It must be pointed out that the initial water saturation for the homogeneous case was iteratively tuned to reach a suitable history match, which was uniform in the whole domain, as mentioned before. On the contrary, the heterogeneous case did not require any tuning. In this case, the model only relied on the measured data from the well logging.

**Fig 3:**
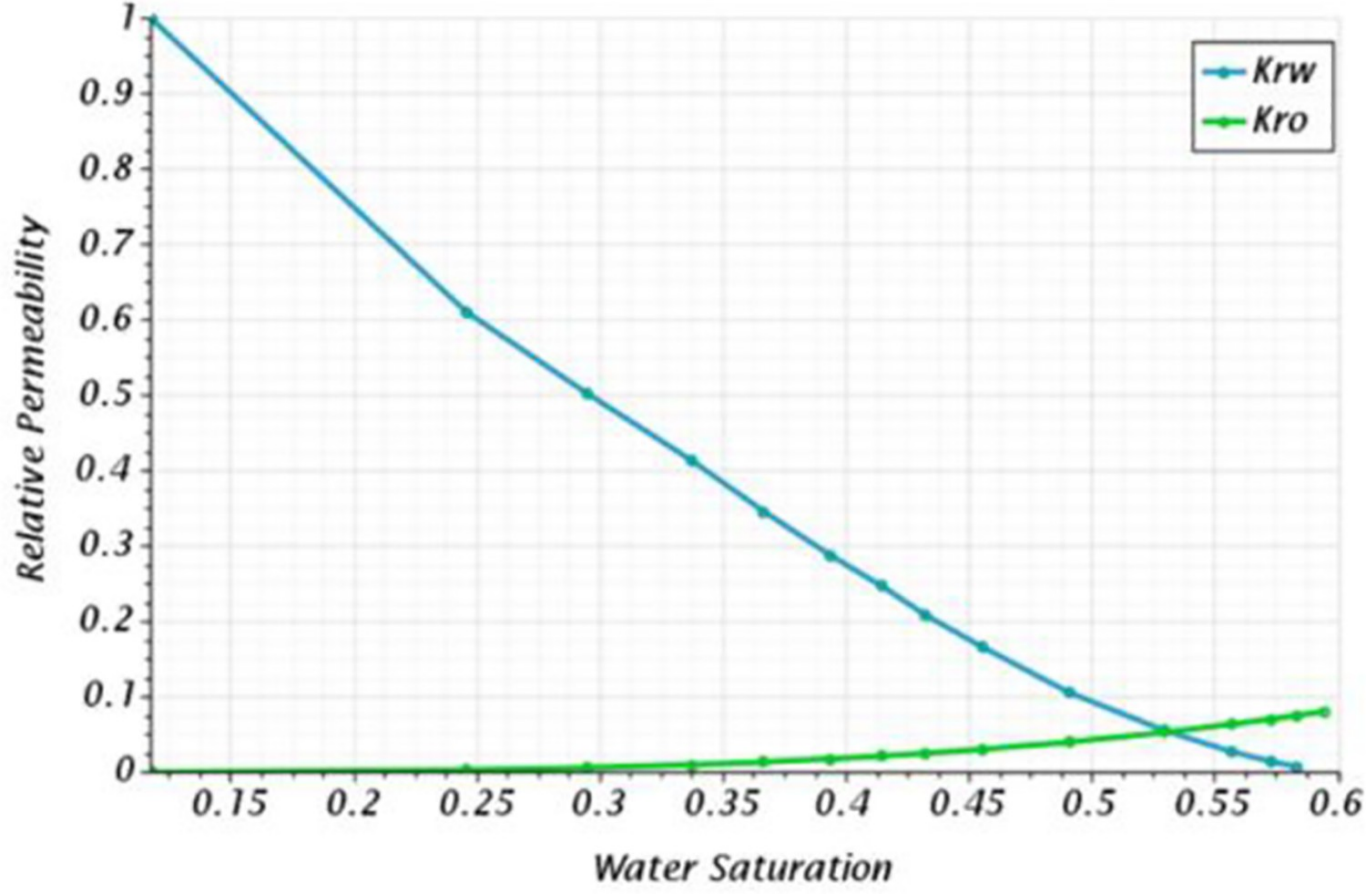
Relative permeability curve measured from the core analysis of the studied reservoir.

In both cases, the domain is divided into three main regions, the reservoir rock, the completion liner, and the well. The reservoir rock in the CFD model had the porous properties previously presented in Table 3 and Fig 2. The completion liner was simulated as a porous region with the concentric flow to reduce the computational costs of modeling the liner’s slots, which is impossible in such a domain considering the large number of slots per foot. Additionally, it was imperative to consider the relative permeability profiles to model multiphase flows in porous media. Fig 3 presents the relative permeability curve for the studied reservoir obtained from core analysis. This curve was considered in the CFD code to model multiphase flow in porous media, as the behavior of one phase relative to the other will depend on their saturation.

### Modeling of the heavy oil reservoir

This subsection emphasizes the main physical models used to simulate the unstable multiphase flow in porous media and the main assumptions, like modeling the slotted liner as a porous region instead of the slots’ explicit modeling.

#### Modeling of the slotted liner

**Fig 4:**
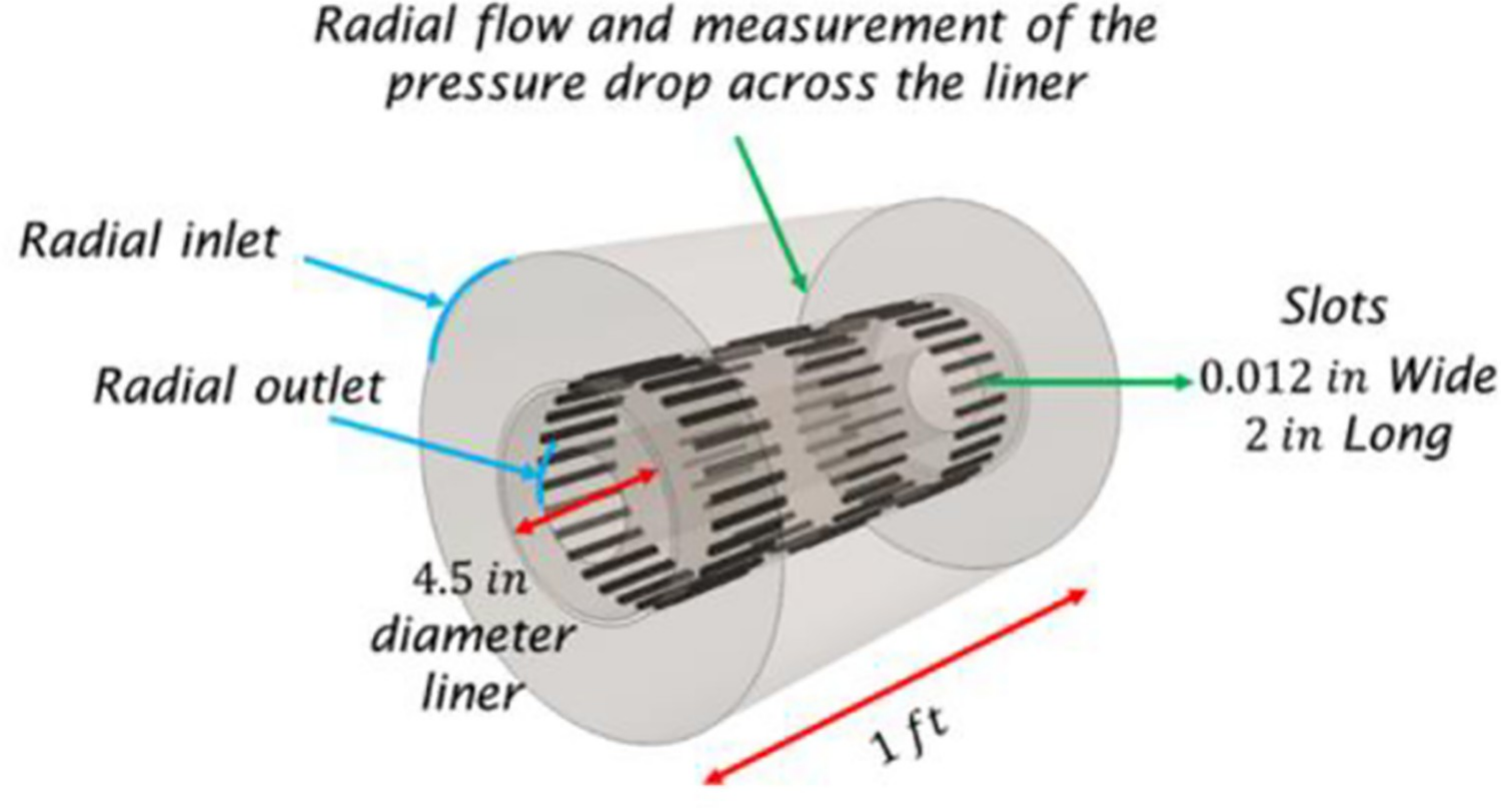
Schematic of the small section of the completion liner used to calculate the viscous and inertial resistance to simulate this liner as a porous region.

The liner slots’ explicit modeling was computationally impossible, especially for 224 slots per foot design. Therefore, one of the major assumptions to overcome this issue was to model the completion liner as a porous region. Therefore, it was necessary to determine the liner’s viscous and inertial porous resistances, which were made from CFD simulations due to the inexistence of experimental or technical reported data in this regard. Fig 4 presents a brief schematic of the CFD model developed to calculate a slotted liner with a staggered pattern design.

Calculating the viscous and inertial porous resistances consisted of measuring the pressure drop across the liner for different flow rates. The flow entered from the outer casing, shown in Fig 4, going radially towards the center of the system, passing through the liner and exiting in the center of the domain. The pressure drop was measured by placing pressure sensors outside and inside the liner walls until a steady-state reading was obtained. Then, the porous resistances for oil and water were calculated using a mathematical regression from the pressure drop profile against flowrate, satisfying the Dupuit-Forchheimer equation, which describes the porous media flow, presented in Eq 1.


$$Ƥ={Ƥ}_{v}+{Ƥ}_{i}|v_{s}|$$ Eq 1

Where Ƥ is the porous resistance tensor, Ƥ_*v*_ the linear viscous resistance tensor, Ƥ_*i*_ the quadratic inertial resistance tensor, and *v*_*s*_ the flow velocity. Finally, the results obtained from this process are presented in Table 4.

**Table 4:** Results for the calculation of the inertial and viscous porous resistances for the slotted liner.

Flow (*m*/*s*)	Pressure drop (*Pa*)	
	Oil	Water
0.2	131961.14	12399.75
0.4	357141.94	48913.29
0.6	676461.52	194385.20
0.8	1090772.78	436436.10
**Calculated results for the viscous and inertial resistances**		
Ƥ_*v*_(*kg*/*m*^3^*s*)	7.03*x*10^7^	6.17*x*10^5^
Ƥ_*i*_(*kg*/*m*^4^)	1.95*x*10^8^	2.00*x*10^8^

#### Discretization of the domain

**Fig 5:**
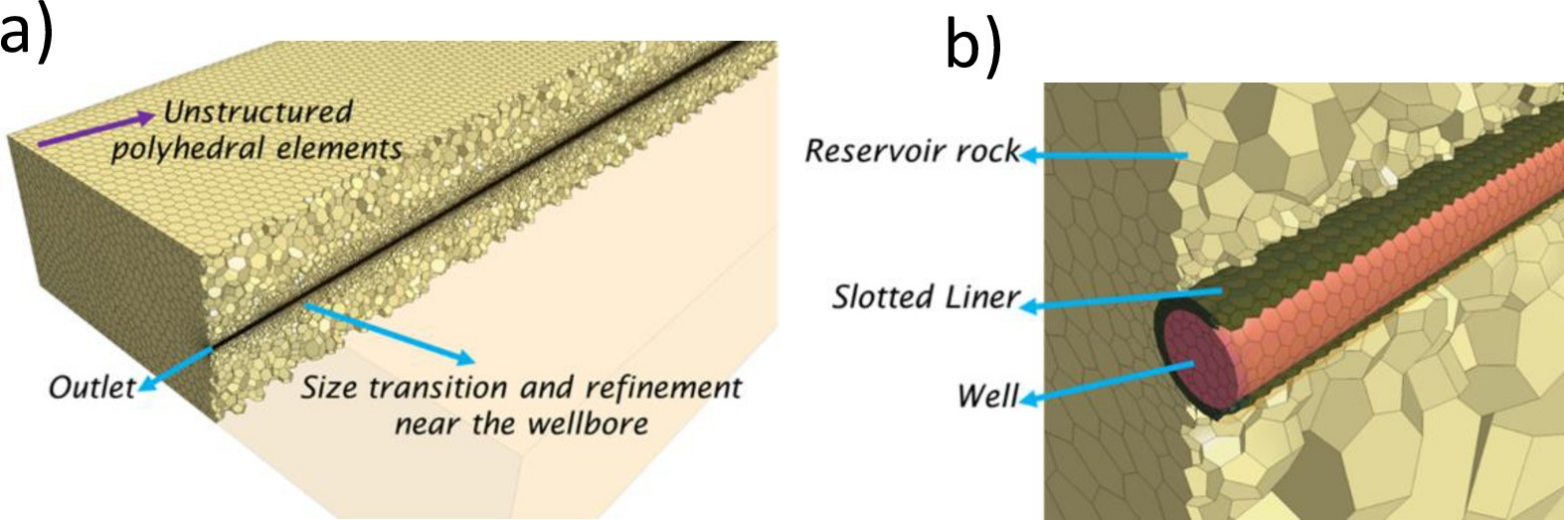
Representation of the mesh used in this study, a) general view, b) focus on the well and liner regions.

A previous study proposed a methodology for simulating unstable displacements considering VF at the continuum scale based on CFD models [53]. This methodology suggested an average Courant-Friedrichs-Levy (CFL) number of 0.25 for the flow measured in the reservoir rock with fair agreement against experimental data for oil recovery, along with the qualitative description of the VF dynamics and topologies in 3D. Therefore, the mesh size and time-step were scaled up to satisfy this numerical constraint in the current study. As a result, a time-step of 1200*s* and the unstructured polyhedral mesh described in Table 5 and Fig 5 were used. These discretization parameters can describe the displacement of the bottom aquifer, where most of the mesh is dedicated to the reservoir rock. Although the spatial discretization becomes too coarse to simulate the VF dynamics explicitly, it can describe the aquifer’s strong channeling in great detail.

**Table 5:** Element count per region obtained for the meshing of the domain using unstructured polyhedral elements.

Region of the domain	Element count
**Well**	158451
**Liner**	274458
**Reservoir rock**	487789

#### Physical models and conservation equations

The mathematical models on which the CFD code used is based, STAR-CCM+ v15.04, are the continuity, momentum, and energy conservation equations. The Eulerian-Eulerian Volume of Fluid (VOF) model is considered to simulate the immiscible multiphase flow of heavy oil and water. Also, it uses the High-Resolution Interface Capturing Scheme to track the unstable interface. The CFD model also considers gravity for phase segregation and as an external force that competes against the viscous forces. The detailed mathematical formulation is presented in S1 Apendix.

The numerical model scheme was the co-located variable arrangement to solve the conservation equations on the discretized domain. Conversely, the conservation equations’ surface terms are evaluated using quadrature approximations with second-order accuracy. While the volume terms are evaluated at the cell center using second-order accuracy too. Moreover, it must be mentioned that the convective flux terms are solved using second-order upwind schemes. Simultaneously, the diffusive flux also uses a second-order scheme based on the decomposition of the gradient terms. These gradients are calculated using the Hybrid Gauss-Least square method, which uses blending factors to determine which method will be used. For example, when it assigns a blending factor of one, it uses the least square method, while it uses the Green-Gauss method on a blending factor of a cero. Finally, the CFD code uses a segregated solver based on the SIMPLE algorithm to couple the pressure and velocity results to solve the conservation equations numerically.

## Results

This section presents the most relevant quantitative and qualitative results about the sensitivity analysis, the verification of the numerical model, and the comparison of the displacement dynamics near the wellbore for the homogeneous and heterogeneous study cases.

### Influence of different fluid and homogeneous rock properties on the severity of water production

This section addresses the influence of different fluid and rock physical properties over the heavy oil-water displacement near the wellbore. This analysis also determines the feasibility of using CFD codes to model heavy oil reservoirs. Such numerical studies have not been reported before and serve as an alternative to conventional reservoir codes. The analysis considers parameters such as permeability, porosity, viscosity ratio, density ratio, and initial water saturation. Additionally, this sensitivity analysis is conducted considering a homogeneous rock and a constant production rate of 1000 *bbl*/*d*, to keep the analysis as comparable as possible. The fluid and rock physical properties are the same ones previously presented in Tables 1 and 2.

#### Analysis of the porosity and absolute permeability

The study of the influence of the rock physical properties has mainly addressed the heterogeneity of the porous rock either by experimental [54–56] or numerical [39,47,57–59] means. This subsection analyzes the influence of the rock porosity and permeability, separately, on the displacement dynamics, breakthrough event, and production profiles in horizontal wells. First, Table 6 summarizes the results obtained for the breakthrough time, which for every case occurred near the heel with the subsequent formation of a cresting. In this study, the breakthrough time refers to the amount of time it takes the aquifer to reach the production well once the fluids move in the porous rock. The breakthrough time was calculated by tracking the interface between the aquifer and the oil layer and computing the time it took to reach the horizontal well.

**Table 6:** Calculated breakthrough times for the porosity and permeability sensitivity analysis.

Parameter	Value	Breakthrough time (*hr*)
**Porosity (%)**	10	63.00
	20	126.25
	30	190.00
	40	251.25
	50	320.00
**Permeability (*d*)**	0.01	216.25
	0.1	208.75
	1	166.25
	10	66.00
	100	26.00

The porosity analysis was characterized by a significant variation on the breakthrough time, where a linear relationship was found. For the studied conditions, at lower porosities, the breakthrough time tends to decrease. This behavior is explained because the flow area increases at higher porosities, causing a decrease in velocity and increase in pressure, satisfying the flow energy conservation equation or Bernoulli’s Theorem. Therefore, the fluids move faster, causing an earlier breakthrough compared to their highly porous counterparts.

On the other hand, significant differences in the displacement dynamics were not found. Either during the initial ascend of the aquifer or near the wellbore, where the increase in mesh density could have captured some differences due to the enhancement of the interface resolution. Finally, the shape of the cresting, along with the breakthrough location, remained almost invariant. However, a further numerical study is suggested to confirm these results with a different formulation of the momentum equation. For example, it was considered a model based on the physical velocity rather than superficial velocity to simulate the porous media velocity field. At least to confirm the linear relationship.

For the permeability analysis, the results were somewhat different. The permeability refers to the conductivity of the porous rock. It tells the rock’s ability to allow the flow of fluids, where the highest permeabilities indicate that the fluids can flow easier than low permeability rocks. Therefore, high permeabilities will favor the interface disturbance, fomenting the unstable displacement of the water, oil bypassing, and earlier water breakthrough as reported experimentally [55]. The CFD model could emulate this behavior according to the results presented in Table 6, where the highest permeabilities caused the earliest breakthrough times. Moreover, contrary to the porosity analysis, the profile of breakthrough time was not linear. For example, for an increase in permeability 1*x*10^4^ times, the breakthrough event was 8.3 times faster. On the other hand, the displacement was characterized by a noticeable change of the aquifer movement where a cresting tends to happen at permeabilities below 1*d*. Above this threshold, the displacement’s stability worsened near the heel, where a strong channeling tends to occur without forming a crest. Instead, sharp and well-defined conning at the heel happened.

**Fig 6:**
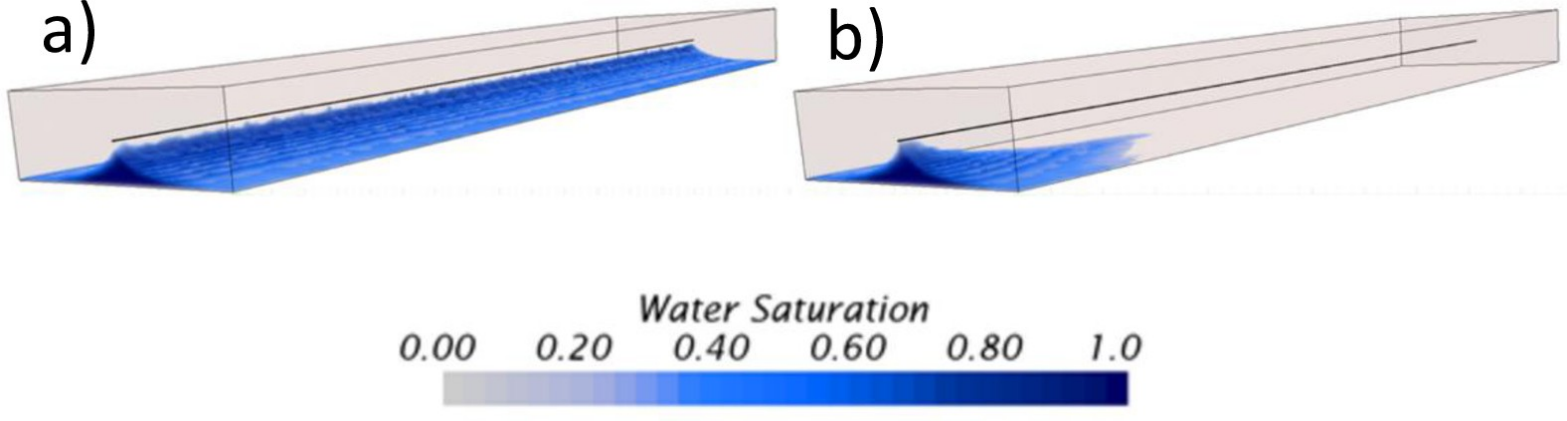
Dynamics of the water movement near the wellbore for the permeability sensitivity analysis, a) at *k* = 0.01*d*, b) at *k* = 100*d*. 3D renders taken at the breakthrough time.

Fig 6 describes the three-dimensional profile of the aquifer movement near the wellbore at the breakthrough time. The blue color refers to the water, while the transparent color above the interface refers to the heavy oil to emphasize the aquifer movement. This figure shows the mentioned differences between both permeabilities. The lowest permeability, 0.01*d*, had a well-defined cresting. Moreover, due to the enhancement of the interface resolution near the wellbore caused by the increase of mesh density, the model predicted the formation of small channelings during the breakthrough event along the well. In contrast, the aquifer in the case of 100*d* had a rapid ascent near the heel, with a well-defined conning instead of a crest.

**Fig 7:**
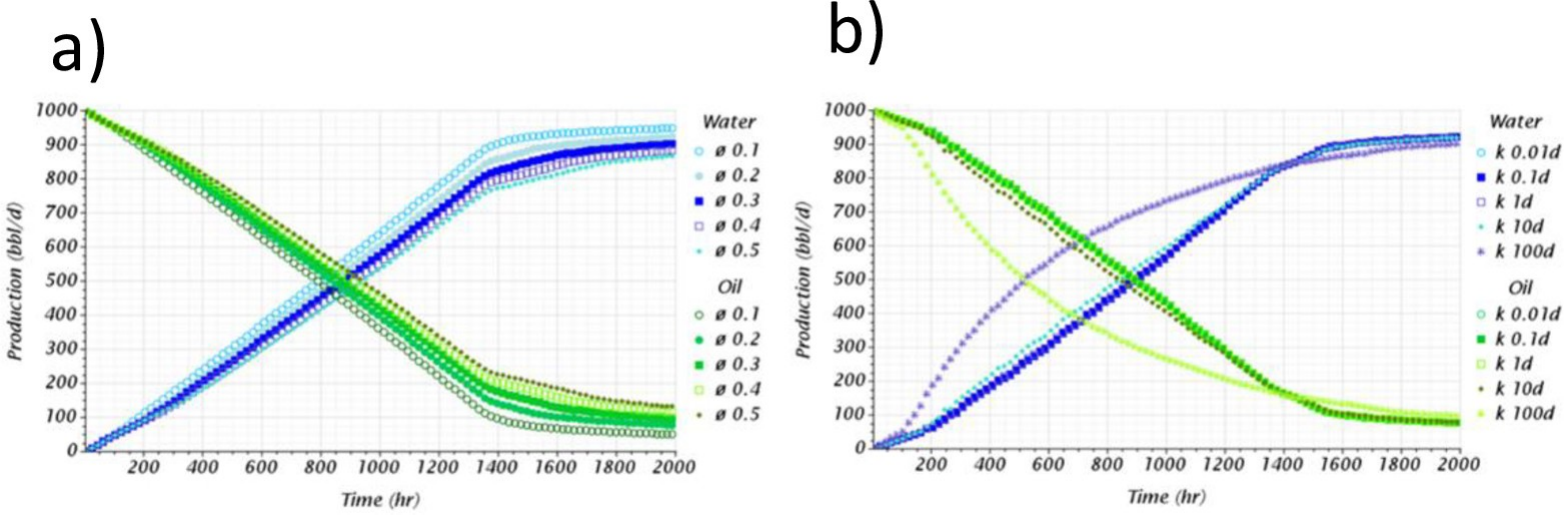
Production profile obtained for the a) porosity and b) permeability sensitivity analysis.

On the other hand, the production profiles presented in Fig 7 reflect the influence on these two parameters’ variation. For the porosity case, the production variations are favored by the increase in porosity where lower water-cuts were obtained at the end of the simulation time. Again, the physical explanation of this behavior is due to the reduction of the flow area at low porosities explained earlier. The profiles exhibit a sharp increase and decrease in water and oil production, respectively, with an almost linear constant slope. Finally, the porosity sensitivity analysis indicated that it could produce more oil in highly porous systems. For example, between the cases with a porosity of 10 and 50%, it was determined that it was possible to produce almost three (3) times more oil per day in the 50% porosity case.

To conclude, the production profiles for the permeability sensitivity analysis trend to remain almost invariant between the cases with a permeability of 0.01 and 1*d*. The last two cases, 10 and 100*d*, the highest permeabilities studied, showed different production profiles, especially the 100*d* case. Contrary to the porosity analysis, the breakthrough event is reflected by the slope change that can be distinguished at the beginning of the production time in these profiles, close to 200*hr* in most cases. However, the 100*d* case showed the highest difference by having the fastest breakthrough time, 8.3 times faster than the lowest permeability case. Finally, it is also noticeable how the large permeability cases had a rapid and dramatic increase in water production, with the unfortunate reduction of oil in the same proportion, especially for the 100*d* case.

#### Analysis of the oil viscosity and density

The fluids’ physical properties, like the viscosity ratio, play a significant role in displacement stability. The viscosity ratio in the present study refers to the relationship between the viscosities of the displaced and displacing fluids, i.e., the relationship between the viscosities of the heavy oil and water (*μ*_*r*_ = *μ*_*oil*_/*μ*_*water*_) Several experimental studies in Hele-Shaw cells and porous media have demonstrated that increasing the viscosity ratio worsens the stability, causing earlier breakthroughs and reducing the oil recovery [5,54,55,60]. The numerical results obtained from the viscosity sensitivity show the same trend, demonstrating that CFD codes can emulate this natural behavior. On the one hand, the results from Table 7, which summarize the calculated breakthrough time, indicate that increasing the viscosity ratio reduces the breakthrough time dramatically. For example, it was determined that increasing the viscosity ratio from 0.001 to 0.01, i.e., ten times, rushed the breakthrough 1.2 times faster. In contrast, for a viscosity ratio of 1*x*10^5^, the consequences worsened as the water breakthrough was 9.6 times faster than the lowest viscosity ratio case.

**Table 7:** Calculated breakthrough times for the oil viscosity and density sensitivity analysis.

Parameter	Value	Breakthrough time (*hr*)
**Oil Viscosity (*Pa*∙*s*)**	0.001	256.25
	0.01	215.25
	0.1	206.00
	1	153.75
	10	66.66
	100	26.66
**Density (*kg*/*m*** ^**3**^ **)**	992.02	183.33
	978.04	
	965.05	
	952.06	
	939.08	
	927.09	

**Fig 8:**
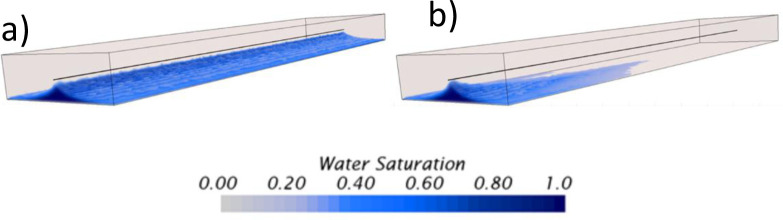
Dynamics of the water movement near the wellbore for the viscosity sensitivity analysis, a) at μ_oil_ = 0.001 Pa∙s, b) at μ_oil_ = 100 Pa∙s. 3D renders taken at the breakthrough time.

**Fig 9:**
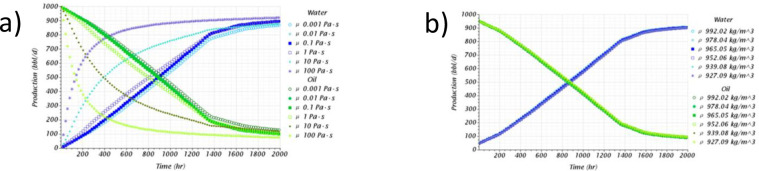
Production profile obtained for the a) viscosity and b) density sensitivity analysis.

The CFD model was also capable of emulating the unstable displacement in the reservoir rock, as presented in Fig 8. This figure compares the water movement for the viscosity ratios of one (1) and 1*x*10^5^ at the breakthrougth time. For example, while Fig 8A exhibits a well-defined crest for the viscosity ratio of one (1), Fig 8B shows a different pattern for the viscosity ratio of 1*x*10^5^. In this case, the flow pattern is characterized by a sharp water conning near the heel. Finally, this conning, which is smaller in terms of the overall size compared to the cresting as it does not propagate along the horizontal well, is capable of channeling larger amounts of water. This is reflected from the production profiles presented in Fig 9, where the water production is 1.3 times higher for the viscosity ratio of 1*x*10^5^ than the viscosity ratio of one (1). For example, for the simulated time, while the total water production was about 3.43*x*10^9^ *bbl* for the lowest viscosity ratio, the total water production for the highest viscosity ratio was of 4.48*x*10^9^ *bbl*, 30% more in comparison.

Displacements with large density contrasts can cause gravity segregation between the phases. However, this feature has been observed for horizontal displacements, causing oil recovery issues [61–63]. The density sensitivity analysis results did not show a significant difference in oil recovery in the present study. Either for the displacement stability near the wellbore for the aquifer vertical upward displacement. For example, the results previously presented in Table 7 indicated that the density contrast did not affect the breakthrough time.

Moreover, the production profiles also remained invariant according to the results of Fig 9B. Therefore, it could be concluded that the density ratio does not affect the displacement stability or the oil recovery, at least for a homogeneous porous media at constant drawdown. However, horizontal displacements suggest further study where gravity segregation can influence the phases’ movement in the porous media.

#### Analysis of initial water saturation

The initial water saturation, *S*_*wi*_ is a parameter that can influence oil recovery. Experimental and theoretical studies have shown that in water-wet systems, the oil recovery decreases at increasing initial water saturation [64–67]. The initial water saturation, at least for the studied conditions, mildly influenced the breakthrough time, as presented in Table 8. It was observed a maximum deviation of 17% between the most spread cases, *S*_*wi*_ = 0% and 30%. Moreover, it was found that the breakthrough time progressively decreased at increasing initial water saturations up to an inflection point, in this case, between an *S*_*wi*_ = 30% and 40%. Displacement fronts have been reported to have a non-monotonic velocity profile as function of the initial water saturation, as experimentally reported by Bauters et al. [68] on the study of Viscous Fingering. Therefore, it is expected that the prediction of the breakthrough to have similar behavior, which the numerical model emulated.

**Table 8:** Calculated breakthrough times for the oil viscosity and density sensitivity analysis.

Parameter	Value	Breakthrough time (*hr*)
**Initial Water Saturation (%)**	0	176.66
	10	170.00
	20	153.33
	30	146.66
	40	156.66

**Fig 10:**
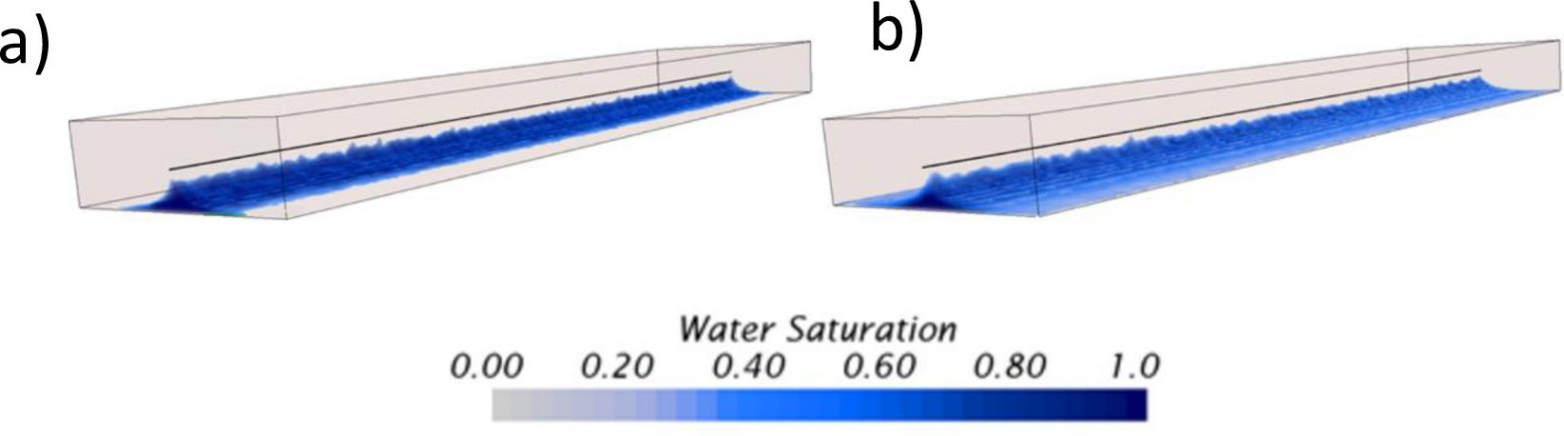
Dynamics of the water movement near the wellbore for the S_wi_ sensitivity analysis, a) at S_wi_ = 0%, b) at S_wi_ = 40%. 3D renders taken at the breakthrough time.

Fig 10 reports the movement of the bottom aquifer for the *S*_*wi*_ = 0 and 30% cases at the breaktrougth time. The displacement near the wellbore was noticeably affected by the initial water saturation in terms of the crest’s widening. The crest for the *S*_*wi*_ = 0% case, presented in Fig 10A, is characterized by being sharper and narrower, occupying a minor cross-sectional area at the bottom of the reservoir compared to its counterpart. On the contrary, the wide of the crest is larger for the *S*_*wi*_ = 30% case, which rises occupying the complete cross-section, as shown in Fig 10B. This change in the flow patterns is attributed to a transition to a diffusion-like regime at increasing initial water saturations, causing a wider and broader water front. This feature has been characterized at the microscale on experimental and numerical studies of Viscous Fingering [68,69]. At low initial water saturations, the displacements are characterized by well-defined fingering patterns. However, the fingers grow slower and wider at fully saturated conditions, disappearing in a diffusion-like displacement front, as pointed out by Kmec et al. [69].

**Fig 11:**
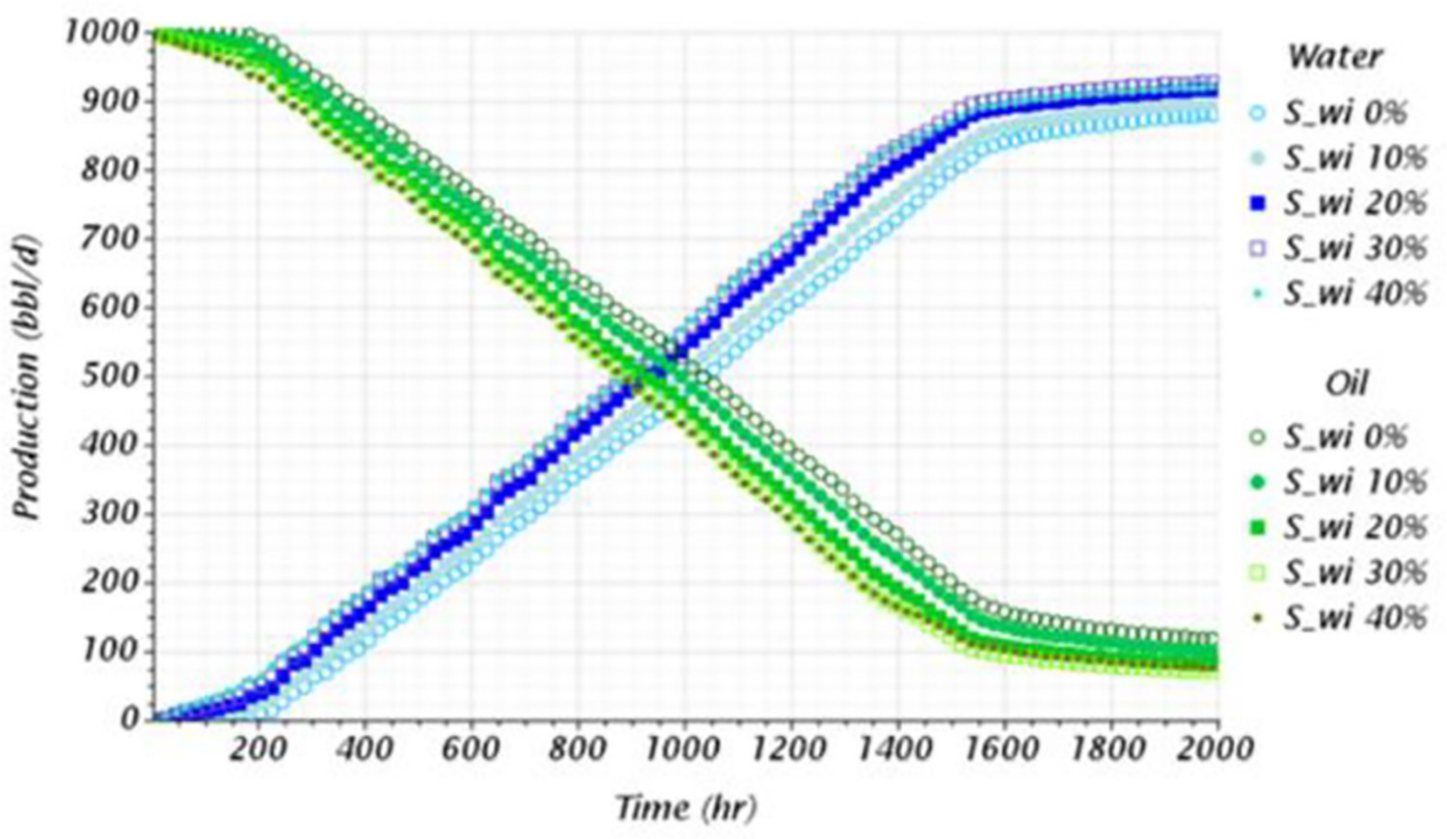
Production profile obtained for the *S*_*wi*_ sensitivity analysis.

Finally, Fig 11 shows the production profiles where the oil production decreases at increasing initial water saturations. Comparable behavior to the experimental results reported in the previously mentioned literature [64–67]. Moreover, after comparing the cases with an *S*_*wi*_ = 0% and 40%, the accumulated oil production decayed from 1.06*x*10^6^ *bbl* to 9.13*x*10^5^ *bbl*, a reduction of 13.86%. Achieving a lower oil recovery is attributed to the minor amounts of the non-wetting phase in the reservoir rock. Therefore, with lower amounts of oil in porous media, it is reasonable to obtain lower production.

### Influence of considering homogeneous and heterogeneous rock properties on the verification of the model

**Fig 12:**
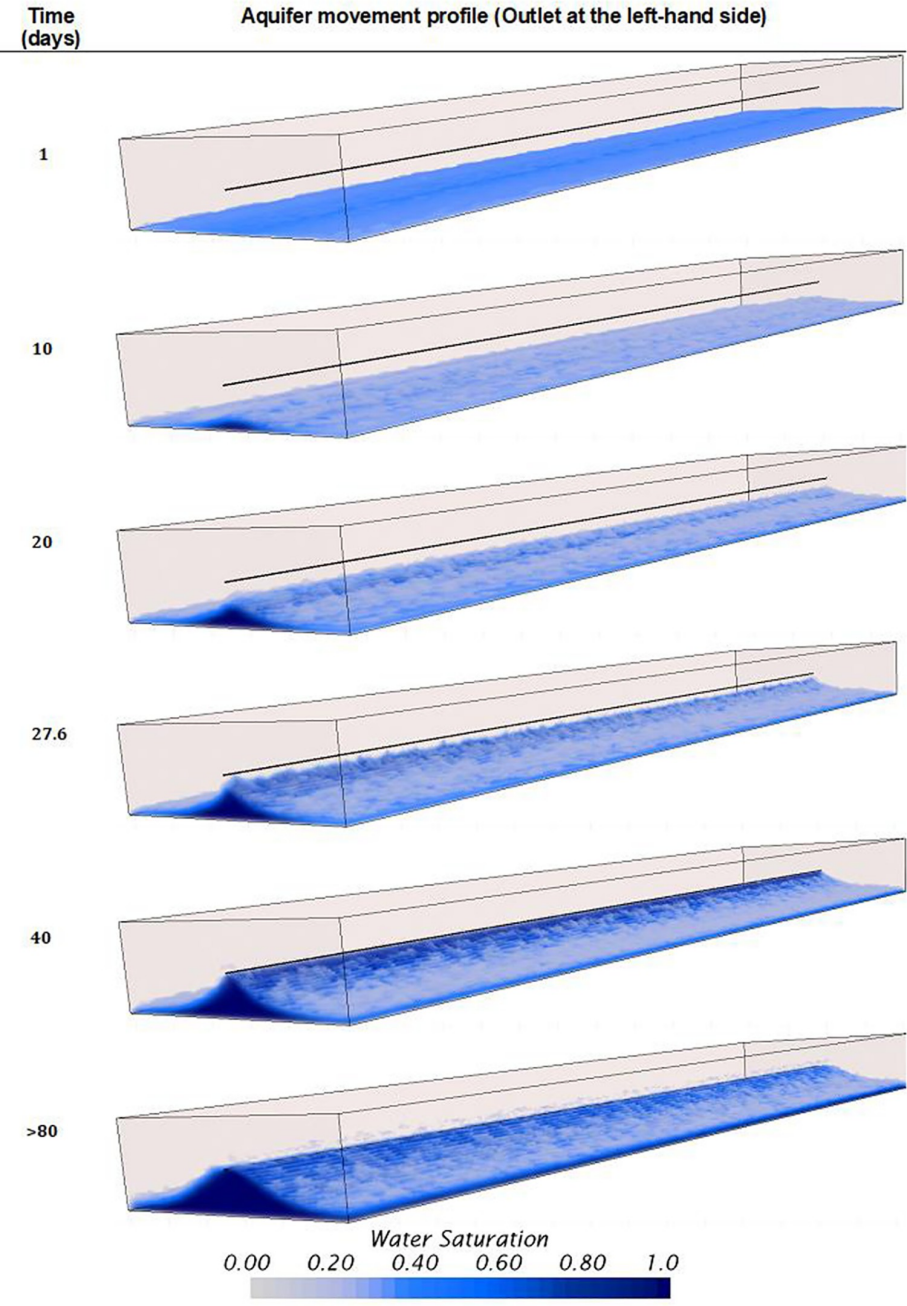
Aquifer movement profile obtained or the homogeneous model.

**Fig 13:**
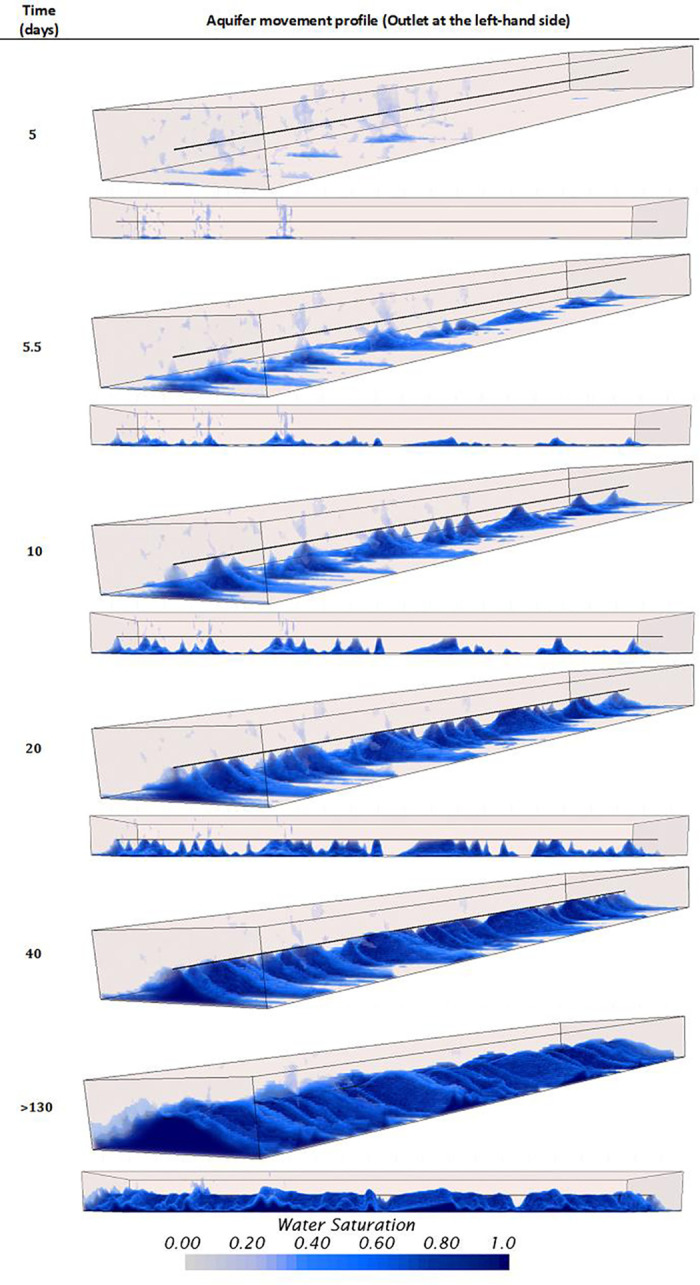
Aquifer movement profile obtained or the heterogeneous model. General view at the top, lateral view at the bottom.

This section provides quantitative verification or validation that CFD numerical models can simulate unstable displacements of heavy oil horizontal wells with bottom aquifers. A comparison between homogeneous and heterogeneous rock properties is conducted, showing the noticeable differences that can be achieved using these numerical codes, offering a high level of detail about the aquifer’s displacement dynamics. Figs 12 and 13 presents the 3D profiles of the water movement for the homogeneous and heterogeneous cases, respectively.

For the homogeneous case, the displacement is unstable as the aquifer rises faster below the well during the first 10 days. After this time, the interface is disturbed, especially near the heel, where a sharp crest is now noticeable. Moreover, a well-defined crest arises along the horizontal section. The explanation of this behavior is attributed to the pressure gradients along the well, where the most significant gradients between the wellbore and the reservoir are near the heel rather than the toe. In this case, the average pressure near the well heel and toe, before the breakthrough, was 1134.46 and 1135.62*psi*, respectively.

From the prediction of the aquifer movement, it was possible to calculate the breakthrough event too. In this case, it was calculated to happen after the first 27.6 *days* of production, strictly at 4.72*ft* from the heel. The breakthrough event location was calculated thanks to the resolution capabilities achievable with the CFD model, especially near the wellbore due to the mesh density increase. Even though the formation and rise of an almost uniform crest was calculated, it was possible to predict the appearance of small isolated channelings near the wellbore. Moreover, it was possible to predict that the crest’s propagation from this initial breakthrough location did not occur. Instead, numerous channelings along the wellbore appeared simultaneously, indicating breakthroughs on several locations during the same timespan. It is suggested to confirm this finding by solving the water moves up to the VF scale by implementing adaptive mesh methodologies at the interface [70,71]. It is possible that near the wellbore shielding mechanisms cause the non-uniform propagation of the cresting, as suggested in this study.

For the heterogeneous case, the results were completely different. Here, the displacement’s stability was strongly influenced by the vertical heterogeneous field of the oil reservoir. Contrary to the homogeneous case, the significant difference is that the cresting did not happen, even beyond the breakthrough event. Instead, strong channeling at different sections near the wellbore characterized the displacement. Moreover, it was possible to identify that the initial breakthroughs occurred in the sections with the highest initial water saturation, above 0.4, and not at the heel as in the homogeneous case. Fig 13 shows the movement of the aquifer, where water pockets can be easily identified in the domain even before the well started production. The aquifer prefers to ascend in regions with high initial water saturation rather than high permeability zones when contrasting the results against the heterogeneous field previously presented in Fig 2.

The prediction of the breakthrough event also differed considerably between both cases. In the heterogeneous case, the initial breakthrough occurred at 132.53*ft* from the heel, not near the heel as in the homogeneous case. Moreover, the breakthrough time occurred at 5.5*days*, 4.6 times faster than the homogeneous case. The subsequent breakthroughs occurred at several locations along the well, nevertheless, without forming a well-defined cresting. Even more, the cresting never happened, contrary to the homogeneous case, which already occupied the wellbore after 40*days*, as showed in Fig 12. On the contrary, for the heterogeneous case, even after 120*days* there were some sections along the well where the aquifer did not ascend. Sections with the lowest initial water saturation and permeability, according to the contour profiles presented in Fig 2B and 2C.

**Fig 14:**
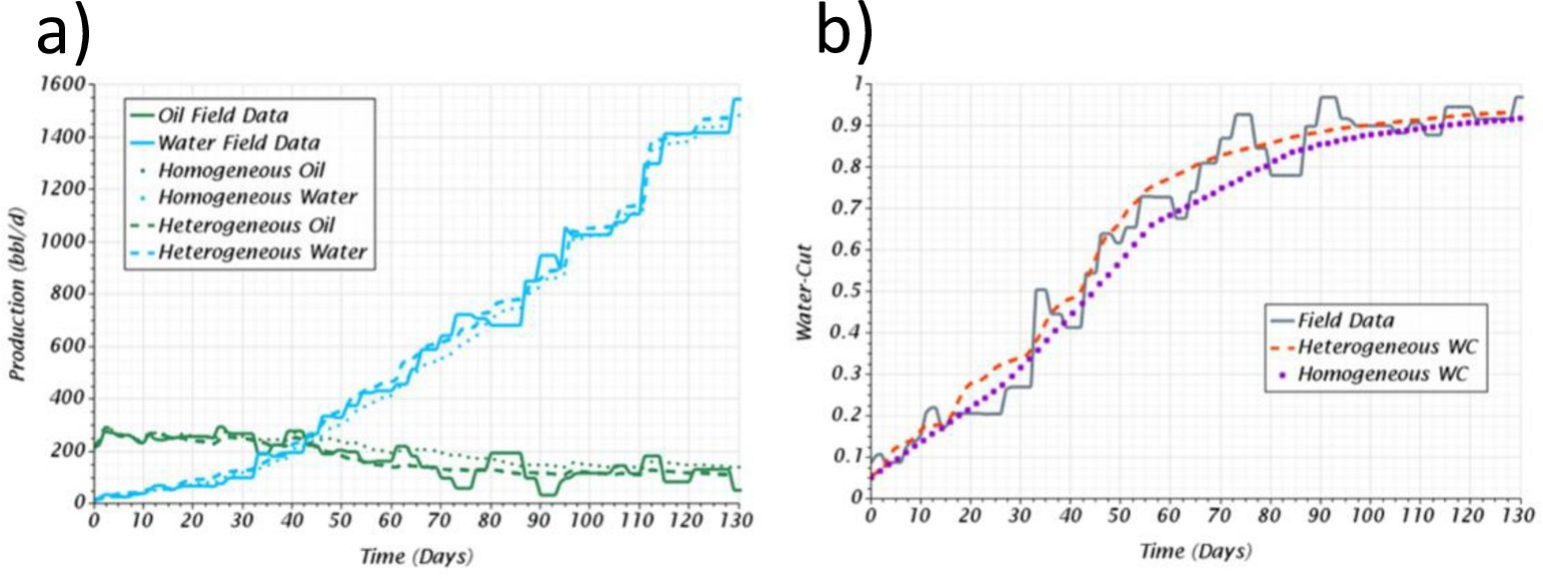
Results of the history match for the homogeneous and heterogeneous case, a) comparison against the reported oil and water production, b) comparison against the reported water-cut.

A history match was conducted to verify or validate the numerical model for both the heterogeneous and homogeneous cases. The validations were made with the field data for a reasonable timespan that considered the well startup, the breakthrough event, and the severe water production after reaching a water-cut of 90%. The validation results for both models are presented in Fig 14 for the production profiles and the water-cut. These profiles demonstrate that the CFD model can reproduce the same production trend in both cases, where the first 41 *days* had a good agreement with a mean absolute error of 31 and 16% for the homogeneous and heterogeneous case, respectively, considering both water and oil production for the calculation of the numerical error. A slightly larger deviation was calculated for the latter days, about 43 and 19% for the homogeneous and heterogeneous cases, respectively. This larger deviation could be attributed to uncertainties related to the field data, such as the sharp jumps that were not considered in the numerical model or treated or eliminated from the numerical error calculation.

Table 9 presents and summarizes the overall mean absolute error, where the deviation for the heterogeneous case was less than twice compared to the homogeneous case. According to these results, the CFD model’s verification could be considered a success, at least for the heterogeneous case. The numerical model based on CFD can qualitatively describe the aquifer movement and predict the production profiles with fair agreement against field data. Additionally, it was determined that the heterogeneous model required less effort than the homogeneous one, which needed the iterative tuning of the initial water saturation to achieve fair quantitative results. Although other parametric adjustments could be made on the homogeneous case, for example, the tunning of the permeability, the heterogeneous could be considered more reliable as it does not need these adjustments.

**Table 9:** Mean absolute error between the numerical results and the field data.

Case	Production data	Mean absolute error (%)	Total mean absolute error for each case (%)
**Homogeneous**	**Oil**	32.13	39.44
	**Water**	46.74	
**Heterogeneous**	**Oil**	25.01	18.39
	**Water**	11.68	

Beyond the information about predicting the aquifer’s unstable displacement and the production profiles through time, the numerical model can give significant results about the horizontal well’s hydrodynamics. Here it will briefly describe the heterogeneous case results using the animations provided in the supplementary material. These results are about the behavior of the pressure (S1 Fig), velocity (S2 Fig), and even the mixture viscosity (S3 Fig) inside the well through time, which can be helpful information to understanding heavy oil reservoirs with horizontal wells. Further supporting information related to the production and water-cut througth time can be found in S4 and S5 Figs, respectively.

For example, the animations about the velocity profile inside the well showed a progressive increase in the mean velocity in the well for two reasons. The first is the increase in production, which consequently increases the mean velocity, and the second is due to the increase in the water-cut. The increase in the water-cut inside the well necessarily affects the mixture viscosity. A higher concentration of the water phase means a decrease in the mixture viscosity, reducing the flow resistance achieving higher velocities inside the well. The animation of the mixture viscosity supports this. It can be seen the progressive drop of this variable along the well, especially after the breakthrough.

Additionally, the dynamic mixture viscosity profile exhibits disturbances as peaks, illustrating the sudden increase of the water-cut in local zones, attributed to the breakthrough of water at several locations along the well. Finally, the pressure profile animation shows the expected hydraulic behavior in a pipe. This means a significant pressure drop at the beginning of the production, attributed to the high oil content and its large viscosity. While after the breakthrough, the pressure drop is reduced because of the high-water cut, where water has less resistance to flow due to its viscosity, causing a minor pressure drop.

## Conclusions

- A numerical model based on a CFD code capable of simulating the unstable displacement of heavy oil horizontal well with a bottom aquifer is presented for the first time. The model was verified against field data for a heavy oil well in the Llano basin, Colombia, with a mean absolute error of 18% in the oil and water production profile.
- The modeling of producing heavy oil wells based on CFD codes, which uses the full 3D Navier-Stokes equations, is presented as an alternative to conventional reservoir simulators. This method allows a high-resolution description of the fluid’s movement, especially on the water displacement near the wellbore.
- A linear relationship between the porosity and the breakthrough time was found from a porosity sensitivity analysis. A further experimental and numerical study is suggested to confirm this linear relationship which has not been reported before.
- For the studied horizontal production well, it was calculated that the breakthrough event occurred 8.3 times faster for a permeability contrast up to 1*x*10^4^. As reported in the literature, the CFD model could worsen the displacement’s stability at higher permeabilities.
- The total recovered oil is strongly influenced by the viscosity ratio but not the density. In this study, a reduction of 30% in oil production was obtained after comparing viscosity ratios up to 1*x*10^5^. These results validate the CFD model as it could emulate this behavior previously reported in the literature.
- The initial water saturation had noticeable effects on the total oil recovery and the water’s movement near the well. In this study, the oil recovery is reduced at increasing initial water saturations, up to 13%, with a mildly influence on the breakthrough time. These results also serve as validation of the numerical model emulating results previously reported in the literature.

## Supporting information

S1 Fig Pressure profile in the horizontal well. Animation describing the variarion of the pressure profile in the well as consequence of the water movement and breaktrougth trougth time.
(MP4)

S2 Fig Mean velocity profile in the horizontal well. Animation describing the variarion of the velocity profile in the well as consequence of the water movement and breaktrougth trougth time.
(MP4)

S3 Fig Mixture viscosity profile in the horizontal well. Animation describing the variarion of the mixture viscosity profile in the well as consequence of the water movement and breaktrougth trougth time.
(MP4)

S4 Fig Production profile and aquifer movement. Animation describing the production profile through time for oil and water along with the movement of the aquifer.
(MP4)

S5 Fig Mixture viscosity profile in the horizontal well. Animation describing the water-cut profile through time along with the movement of the aquifer.
(MP4)

S1 Appendix Governing equations of the numerical model. Mathematical equations that describes the physical models implemented in the numerical model.
(DOCX)
