# Supplementary material for: CFD study of the water production in mature heavy oil fields with horizontal wells
Source: PLoS One. 2021 Oct 25;16(10):e0258870. doi: 10.1371/journal.pone.0258870 (PMC8544864; doi:10.1371/journal.pone.0258870)
Supplement: S1 Appendix — Mathematical equations that describes the physical models implemented in the numerical model. (DOCX) [file pone.0258870.s006.docx]

**S1 Appendix**

**Continuity equation**

The numerical model implements the Volume of Fluid (VOF) Eulerian physical model coupled to a Porous Media Flow Model based on the superficial velocity formulation, to describe the immiscible multiphase flow in porous media.

The distribution of the phases and the interface is given by the phase volume fraction,$\alpha_{i}$, described in Eqs. A1 and A2. The subscript $i$ refers to the phase$i$. The subscripts $V_{i}$ and $V$ are the volume of the phase $i$ and the volume of the cell, respectively.

| $\alpha_{i}=\frac{V_{i}}{V}$ | (A1) |
| --- | --- |
| $\sum\alpha_{i}=1$ | (A2) |

On the other hand, assumes the material properties treated as a mixtire, as described in Eqs. A3 and A4 for the density $\rho$ and the viscosity$\mu$, respecivelly.

| $\rho=\sum\alpha_{i}\rho_{i}$ | (A3) |
| --- | --- |
| $\mu=\sum\alpha_{i}\mu_{i}$ | (A4) |

A superficial velocity formulation is used to model the flow in porous media. This model is based on the porosity $\emptyset$ and superficial velocity $v_{s}$, which describes the increase of physical velocity when flow enters a porous material, as shown in Eqs. A5 and 6.

| $v_{s}=\emptyset v$ | (A5) | |  |
| --- | --- | --- | --- |
| $\emptyset=\frac{V_{f}}{V}$ | | (A6) | |

Where $V_{f}$is the volume occupied by the fluid, and $V$ is the volume of a cell or control volume.

Eq. A7 describes the continuity equation for multiphase flow in porous media.

| $\frac{\partial}{\partial t}\left( \int_{V} \rho dV \right)+\oint_{A} \rho v\cdot da=\int_{V} SdV$ | (A7) |
| --- | --- |

Where $v$ is the physical velocity, $V$ is the volume, $S$ is a mass source term, and $a$ is the surface area. Combining Eqs. A5 and A6 with A7, the continuity equation for multiphase flow flow in porous media becomes Eq. A8.

| $\frac{\partial}{\partial t}\left( \int_{V} \emptyset\rho\right)dV+\oint_{A} \rho v_{s}\cdot da=\int_{V} S_{u}dV$ | (A8) |
| --- | --- |

**Momentum Equation**

The momentum equation for multiphase phase flow in porous media is given by Eq. A9, where $f_{p}$ is a source term that describes the resistance to the flow imparted by the porous media.

| $\frac{\partial}{\partial t}\left( \int_{V} \rho v_{s} \right)dV+\oint_{A} \rho v_{s}\otimes v_{s}\cdot da=-\oint_{A} PI\cdot da+\oint_{A} T\cdot da+\int_{V} \rho gdV+\int_{V} f_{b}dV+\int_{V} f_{p}dV$ | (A9) |
| --- | --- |

Where $f_{p}$ is the porous resistance tensor, described by Eq. A10.

| $f_{p}=-Ƥ_{v}\cdot v_{s}$ | (A10) |
| --- | --- |

Where $Ƥ_{v}$ is the linear-viscous resistance tensor, which is described in Eq. A11.

| $Ƥ_{v}=\frac{\mu}{k}$ | (A11) |
| --- | --- |

Where$k$ is the absolute permeability.
